# IL-2 Stimulated but Not Unstimulated NK Cells Induce Selective Disappearance of Peripheral Blood Cells: Concomitant Results to a Phase I/II Study

**DOI:** 10.1371/journal.pone.0027351

**Published:** 2011-11-09

**Authors:** Claudia Brehm, Sabine Huenecke, Andrea Quaiser, Ruth Esser, Melanie Bremm, Stephan Kloess, Jan Soerensen, Hermann Kreyenberg, Christian Seidl, Petra S. A. Becker, Heiko Mühl, Thomas Klingebiel, Peter Bader, Jakob R. Passweg, Dirk Schwabe, Ulrike Koehl

**Affiliations:** 1 Pediatric Hematology and Oncology, Laboratory for Stem Cell Transplantation and Immunotherapy, Johann Wolfgang Goethe-University Hospital, Frankfurt, Germany; 2 Institute for Transfusion Medicine and Immunohematology, Red Cross Blood Donor Service, Baden-Württemberg-Hessen, Frankfurt, Germany; 3 Pharmazentrum Frankfurt, Johann Wolfgang Goethe-University Hospital, Frankfurt, Germany; 4 Division of Hematology, University Hospital, Basel, Switzerland; University of Palermo, Italy

## Abstract

In an ongoing clinical phase I/II study, 16 pediatric patients suffering from high risk leukemia/tumors received highly purified donor natural killer (NK) cell immunotherapy (NK-DLI) at day (+3) +40 and +100 post haploidentical stem cell transplantation. However, literature about the influence of NK-DLI on recipient's immune system is scarce. Here we present concomitant results of a noninvasive *in vivo* monitoring approach of recipient's peripheral blood (PB) cells after transfer of either unstimulated (NK-DLI_(unstim)_) or IL-2 (1000 U/ml, 9–14 days) activated NK cells (NK-DLI_(IL-2 stim)_) along with their *ex vivo* secreted cytokine/chemokines. We performed phenotypical and functional characterizations of the NK-DLIs, detailed flow cytometric analyses of various PB cells and comprehensive cytokine/chemokine arrays before and after NK-DLI. Patients of both groups were comparable with regard to remission status, immune reconstitution, donor chimerism, KIR mismatching, stem cell and NK-DLI dose. Only after NK-DLI_(IL-2 stim)_ was a rapid, almost complete loss of CD56^(bright)^CD16^(dim/−)^ immune regulatory and CD56^(dim)^CD16^(+)^ cytotoxic NK cells, monocytes, dendritic cells and eosinophils from PB circulation seen 10 min after infusion, while neutrophils significantly increased. The reduction of NK cells was due to both, a decrease in patients' own CD69^(−)^ NCR^(low)^CD62L^(+)^ NK cells as well as to a diminishing of the transferred cells from the NK-DLI_(IL-2 stim)_ with the CD56^(bright)^CD16^(+/−)^CD69^(+)^NCR^(high)^CD62L^(−)^ phenotype. All cell counts recovered within the next 24 h. Transfer of NK-DLI_(IL-2 stim)_ translated into significantly increased levels of various cytokines/chemokines (i.e. IFN-γ, IL-6, MIP-1β) in patients' PB. Those remained stable for at least 1 h, presumably leading to endothelial activation, leukocyte adhesion and/or extravasation. In contrast, NK-DLI_(unstim)_ did not cause any of the observed effects. In conclusion, we assume that the adoptive transfer of NK-DLI_(IL-2 stim)_ under the influence of *ex vivo* and *in vivo* secreted cytokines/chemokines may promote NK cell trafficking and therefore might enhance efficacy of immunotherapy.

## Introduction

Advanced cell therapy trials with donor natural killer (NK) cells post haploidentical stem cell transplantation (haplo-SCT) provide a promising treatment option for patients with high risk leukemia and tumors. While the established T cell therapies are associated with the risk of graft-versus-host disease (GvHD), NK cells may mediate graft-versus-leukemia/tumor (GvL/T) effects without induction of GvHD. Therefore, immunotherapy with highly purified NK cell donor lymphocyte infusions (NK-DLI) in recipients of haplo-SCT could serve as an attractive alternative cell therapy [1]–[3].

NK cells are key players of the innate immune system, able to distinguish between healthy and malignant cells. NK cell cytotoxicity is mediated by a balance of activating and inhibitory signals [4]. Activating receptors like the natural cytotoxicity receptors (NCR) NKp30, NKp44, and NKp46 and the NK group 2D (NKG2D) receptor trigger cytotoxicity against malignant cells [5]. In contrast, the predominance of inhibitory signals is mediated by killer immunoglobulin-like receptors (KIR) [6]–[8]. Human CD56^+^CD3^−^ NK cells in the peripheral blood (PB) can be subdivided into a major CD56^dim^CD16^+^ population which is highly cytotoxic and a smaller immune regulatory CD56^bright^CD16^dim/−^ population with a potent cytokine producing capacity [9]. In the early phase of reconstitution post SCT, an unusually high percentage of CD56^bright^CD16^dim/−^ NK cells can be determined, which gradually declines in the post-transplant period [10]. A part of these emerging cells are immature with impaired cytotoxic function [11], which makes adoptive donor NK cell immunotherapy post SCT needful to enhance GvL/T effects. To date, first trials and ongoing clinical phase I/II studies show the feasibility of using freshly purified or interleukin-2 (IL-2) activated NK-DLIs for the treatment of high risk patients suffering from leukemia or tumors in both, non-transplant settings and after haplo-SCT as an additional immunotherapy [1]–[3], [12]–[16]. These first immunotherapy trials show that NK-DLIs are infused without immediate adverse events. Moreover, a clinical benefit was reported by Rubnitz *et al.* showing a 2-year event-free survival of 100% for ten children with favorable- and intermediate-risk acute myeloid leukemia (AML) in first complete remission post haploidentical NK cell immunotherapy [3].

However, to date there is a lack of literature concerning studies investigating the influence of allogeneic NK-DLIs on the immune system of the host. Here, we present concomitant data about the noninvasive approach of an *in vivo* monitoring of recipient's cells of the innate and adaptive immune system following treatment with unstimulated in comparison to IL-2 activated NK cells post haplo-SCT. Quantification of various leukocyte subsets together with analysis of cytokine/chemokine plasma levels before and after NK-DLI applications revealed novel information on the immune status of patients undergoing adaptive NK cell therapies.

## Materials and Methods

### Ethics Statement

The study was approved by the Medical Ethics Committee of the Frankfurt University Hospital in 2003 (Ref. number 262/03). Written informed consent was obtained from all children and parents/legal guardians of the children.

### Study design of phase I/II NK cell immunotherapy

Between 2003 and 2011, 16 pediatric patients suffering from high risk leukemia or tumors underwent haplo-SCT and additionally received NK cells from their respective donor (Clin Gov No. NCT 01386619, Table 1+2). For haplo-SCT, peripheral blood stem cells (PBSC) were purified immunomagnetically either by CD34-selection or CD3/CD19-depletion (Clin Gov No. NCT 00945126) as described previously [16], [17]. After haplo-SCT, highly purified donor NK cells were transfused around (+d 3), +d 40 and +d 100 as we described earlier (Fig. S1A+B) [1], [12], [18]. So far, nine patients received highly purified, freshly isolated unstimulated NK cells (NK-DLI_unstim_, group I) and nine patients were treated with further *ex vivo* IL-2 activated NK cells along with their corresponding *ex vivo* secreted cytokines/chemokines (NK-DLI_IL-2 stim_, group II). In two patients both, NK-DLI_unstim_ and NK-DLI_IL-2 stim_ were administered (No. 8 and 9). In summary, 29 NK-DLIs were transfused (n = 15 NK-DLI_IL-2 stim_ and n = 14 NK-DLI_unstim_); of those 14 freshly and 15 following cryopreservation (Table 2 and Fig. S1A+B). According to the study protocol, cryopreservation was an option to verify two to three NK cell applications and reduce the physical loading of repeating leukapheresis for the donors. Targeted cell doses were ≥1×10^7^/kg BW CD56^+^CD3^−^ NK cells, with <1×10^5^/kg BW contaminating CD3^+^ T cells. Defined study exclusion criteria prior to NK-DLI were graft failure or patients with persisting acute or chronic GvHD. Study discontinuation criteria were severe GvHD (≥grade III) or other toxicities.

**Table 1:** Patients' characteristics phase I/II study: NK-DLI post haplo-SCT.

No.	Sex, Age	BW	Diagnosis, Status	Conditioning regime	Graft purification	CD34^+^	CD56^+^ CD3^−^	CD3^+^	GvHD prophy-laxis	GvHD grade	Donor chimerism (PB+BM)		Current state, days post SCT
	[years]	[kg]				[10^6^/BW]	[10^6^/BW]	[10^3^/BW]			d+40 (±10 d)	d+100 (±10 d)	
**Group I: Patients receiving NK-DLI_unstim_**													
1	f, 9	21	ALL, CR2	Flu, Thio, Mel, OKT3	CD34	12.7	0.1	7.1	–	III–IV	CC	CC	†, GvHD,+498
2	m, 15	90	AML, NR	Flu, Thio, Mel, OKT3	CD34	13.6	0.5	7.0	–	–	AR	CC	†, R, GF, +160
3	f, 8	21	M. Hodgkin, PR	Flu, Thio, Mel, OKT3	CD34	29.6	<0.1#	13.5	–	I	CC	CC	†, DP, +126
4	m, 23	50	ALL, CR2	Flu, Thio, Mel, OKT3	CD34	10.3	<0.1#	4.4	–	–	CC	CC	alive, cCR, +2218
5	m, 22	62	RMS IV, NR	Flu, Thio, Mel, OKT3	CD3/19	7.7	12.2	162.1	MMF	IV	CC	CC	†, R , +233
6	m, 10	27	ALL, CR4	Flu, Thio, Mel, OKT3	CD3/19	18.4	17.4	100.0	MMF	II	CC	CC	alive, cCR, +2134
7	m, 9	37	ALL, CR2	Flu, Etop, TBI, ATG	CD3/19	7.0	14.2	<0.5#	MMF	–	CC	CC	†, R, T-DLI, GvHD, Infection, +259
8*	f, 18	49	NB IV, CR2/PR	Flu, Thio, Mel, OKT3	CD3/19	10.0	7.2	96.8	MMF	I	CC	CC	alive, cCR, +742
9*	m, 14	60	AML, NR	Flu, Thio, Mel, OKT3	CD3/19	12.3	10.1	84.5	MMF	I–II	CC	CC	alive, R, +125
Median						12.3	7.2	13.5§					
**Group II: Patients receiving NK-DLI_IL-2 stim_**													
8*	f, 18	49	NB IV, CR2/PR	Flu, Thio, Mel, OKT3	CD3/19	10.0	7.2	96.8	MMF	I	CC	CC	alive, cCR, +742
9*	m, 14	60	AML, NR	Flu, Thio, Mel, OKT3	CD3/19	12.3	10.1	84.5	MMF	I–II	CC	CC	alive, R, +125
10	m, 3	13	NB IV, CR2	Flu, Thio, Mel, OKT3	CD3/19	16.8	61.7	50.0	MMF	I	CC	CC	†, R, +184
11	m, 7	20	NB IV, CR2	Flu, Thio, Mel, OKT3	CD3/19	13.8	13.8	29.4	MMF	II	CC	CC	cCR, +1112
12	m, 8	22	NB IV, PR	Flu, Thio, Mel, OKT3	CD3/19	9.7	12.6	24.9	MMF	I–II	CC	CC	†, EBV ass. B cell lymphoma, +198
13	f, 16	90	ALL, NR	Flu, Thio, Mel, OKT3	CD3/19	8.0	5.8	92.6	MMF	I	CC	CC	†, R, +373
14	f, 16	47	AML, NR	Flu, Thio, Mel, OKT3	CD3/19	8.4	55.8	143.4	MMF	–	CC	MC d+103 (1–5%)	†, R, +245
15	m,15	51	AML, NR	Treo, Flu, Thio, ATG	CD3/19	5.5	25.9	100.1	MMF	–	CC	CC	alive, R, +230
16	f, 1	7	AML, NR	Treo, Flu, Thio, ATG	CD3/19	12.7	17.9	44.4	MMF	–	n.s.	n.s.	†, GF, +27
Median						10.0	13.8	84.5§					

ALL: acute lymphatic leukemia, AML: acute myeloid leukemia, AR: autologous reconstitution, ass.: associated, ATG: anti-thymocyte globulin, BM: bone marrow, BW: kg/body weight, CC: complete chimerism, cCR: continuous complete remission, CD34: CD34 stem cell graft, CD3/19: CD3/CD19 depleted stem cell graft, CR: complete remission, d: days, DP: disease progression, EBV: epstein-barr-virus, Etop: etoposide, f: female, Flu: fludarabine, GF: graft failure, GvHD: graft-versus-host disease, haplo-SCT: haploidentical stem cell transplantation, m: male, MC: mixed chimerism, Mel: melphalan, MMF: mycophenolate-mofetil, NB: neuroblastoma, NK-DLI: NK cell donor lymphocyte infusion, No.: number, NR: non remission, n.s.: not specified, OKT3: Orthoclone/Muronomab, PB: peripheral blood, PR: partial remission, R: relapse, RMS: rhabdomyosarcoma, SCT: stem cell transplantation, T-DLI: T cell donor lymphocyte infusion, TBI: total body irradiation, Thio: thiotepa, Treo: treosulfan,

*patients received both NK-DLI_unstim_ and NK-DLI_IL-2 stim_,

† died,

# under detection limit,

§ difference statistically not significant.

**Table 2:** NK cell applications phase I/II study: NK-DLI post haplo-SCT.

No.	Day of NK-DLI post SCT	fresh (f) cryo (c)	Volume	CD56^+^ CD3^−^	CD56^+^ CD3^+^	CD56^−^ CD3^+^	total CD3^+^	1. KIR MM (GvL/T)	2. KIR MM (GvL/T)	1. KIR MM (HvG)	2. KIR MM (HvG)
			[ml]	[10^6^/BW]	[10^3^/BW]	[10^3^/BW]	[10^3^/BW]	(excluding A3/A11 mismatch)			
**Group I: Patients receiving NK-DLI_unstim_**											
1	+2	c	261	24.7	n.s.	n.s.	53.4	2DL1/C2	3DL1/Bw4	2DL1/C2	–
2	+3	c	499	13.5	n.s.	n.s.	4.5	2DL1/C2	–	3DL1/Bw4	–
3	+2	c	168	32.3	n.s.	n.s.	1.8	2DL1/C2	–	2DL1/C2	–
	+54	f	77	15.5	n.s.	n.s.	8.2				
4	+2	c	154	6.6	n.s.	n.s.	0.8	–	–	2DL1/C2	–
	+49	f	143	12.7	n.s.	n.s.	8.1				
	+103	c	75	3.2	n.s.	n.s.	0.4				
5	+3	c	198	9.9	n.s.	n.s.	0.6	2DL1/C2	–	–	–
	+42	f	115	7.7	n.s.	n.s.	4.1				
6	+2	c	152	6.9	n.s.	n.s.	4.8	–	–	2DL2/C1	–
7	+42	f	92	38.3	n.s.	n.s.	2.3	–	–	2DL2/3/C1	–
	+92	c	60	12.5	n.s.	n.s.	0.8				
8*	+50	f	129	8.7	15.8	<0.6#	15.8	3DL1/Bw4	–	–	–
9*	+47	f	210	30.0	37.6	10.4	48.0	2DL3/C1	3DL1/Bw4	–	–
Median (total)		14	148	13.1	n.s.	n.s.	4.3§§	6/9		6/9	
Median (fresh)		6	122	14.1	n.s.	n.s.	8.2§				
Mean (fresh)			128	18.8	n.s.	n.s.	14.4				
**Group II: Patients receiving NK-DLI_IL-2 stim_**											
8*	+70	c	183	20.6	81.0	17.3	98.3	3DL1/Bw4	–	–	–
	+116	c	187	8.4	31.3	12.4	43.7				
*	+101	f	1155	30.6	18.5	16.1	34.6	2DL3/C1	3DL1/Bw4	–	–
10	+40	f	1000	45.1	50.0	7.5	57.5	2DL1/C2		3DL1/Bw4	–
	+126	c	376	41.4	43.0	6.9	49.9				
11	+35	f	288	7.8	6.0	7.3	13.3	2DL1/C2	–	2DL1/C2	3DL1/Bw4
	+109	c	306	13.5	9.6	13.0	22.6				
12	+39	f	800	19.1	39.5	8.1	47.6	2DL1/C2	–	2DL1/C2	–
	+96	c	148	6.0	22.3	15.3	37.6				
13	+41	f	2296	15.0	29.3	2.4	31.7	2DL2/L3/C1	–	–	–
14	+54	f	319	6.6	8.8	0.3	9.1	–	–	2DL2/3/C1	–
	+96	c	240	6.1	7.3	8.2	15.5				
15	+54	f	684	14.6	11.3	4.1	15.4	2DL1/C2	–	2DL1/C2	–
	+98	c	210	7.3	5.7	2.0	7.7				
16	+11	f	55	14.9	20.9	31.6	52.5	2DL1/C2	–	2DL1/C2	–
Median (total)		15	306	14.6	18.5	8.1	34.6§§	8/9		6/9	
Median (fresh)		8	742	15.0	14.9	7.4	33.2§				
Mean (fresh)			825	19.2	20.2	9.7	32.7				

BW: kg/body weight, cryo (c): cryopreserved, fresh (f): freshly applied, f: female, GvL/T: graft-versus-leukemia/tumor, haplo-SCT: haploidentical stem cell transplantation, HvG: host-versus-graft, KIR: killer cell immunoglobulin-like receptor, m: male, MM: mismatch, No.: number, n.s.: not specified, NK-DLI: NK cell donor lymphocyte infusion, SCT: stem cell transplantation,

*patients received both NK-DLI_unstim_ and NK-DLI_IL-2 stim_,

# under detection limit,

§ difference statistically not significant,

§§ difference statistically significant.

### Purification of CD56^+^CD3^−^ NK cells, *ex vivo* activation and quality control

NK cells were collected from two unstimulated leukapheresis products, without G-CSF stimulation, from healthy haploidentical donors. The two-step purification procedure (CliniMACSs cell selection system; Miltenyi Biotec, Bergisch Gladbach, Germany) included first a CD3^+^ T cell depletion step with an ensuing CD56^+^ NK cell selection from the CD3^−^ fraction, obeying good manufacturing practice (GMP) as we described previously [1], [18]. In case of group I patients receiving NK-DLI_unstim_, the leukapheresis was performed at day −10 prior and +40 post SCT (Fig. S1A). At day +40, NK-DLI_unstim_ was split while one part was applied freshly directly at the end of the purification process, and the other part was cryopreserved for the day +100 application. The processed NK-DLI_unstim_ from day −10 was also split and cryopreserved for the NK cell applications on day +3 and +100. For cryopreservation, NK cell products were concentrated and resuspended in X-VIVO 10 media diluted 1∶2 with 20% dimethyl sulfoxide (DMSO).

In case of group II patients receiving NK-DLI_IL-2 stim_, two leukapheresis products collected on day +29 and +30 post SCT were pooled for the NK cell purification process (Fig. S1B). Following the two-step CD3-depletion/CD56-selection purification procedure, NK cells were further expanded and activated using 1000 U/mL rhIL-2 (Proleukin® Novartis Pharma GmbH, Nürnberg, Germany) for 10 (9 to 14) days obeying GMP. NK cells were cultured in X-VIVO 10 media in VueLife™ cell culture bags (CellGenix Technology, Freiburg, Germany), supplemented with 5% heat-inactivated human fresh frozen plasma (Red Cross Blood Donor Service, Baden-Württemberg-Hessen, Frankfurt, Germany) at 37°C and 5% CO_2_. Fresh media and IL-2 were added every three days. Following the *ex vivo* stimulation, the NK cell product was split up, while one half was infused freshly at day +40 and the other was cryopreserved and applied at day +100 post SCT (Fig. S1B).

For quality control, analyses of purity of NK cells, residual T cells, cell viability, NK cell receptor repertoire (NCRs, NKG2D) and cytotoxic activity against K562 cells were performed [16], [19].

### Sample collection and preparation

Immune reconstitution of various leukocyte subsets in the PB of all patients was monitored regularly; within the first three months post SCT weekly, from month four to six twice a month, followed by a period of monthly analyses. For our concomitant *in vivo* monitoring during NK-DLI, PB samples were collected before (pre), 10 min, 1 h, 4 h and 24 h after the end of NK cell infusion (Fig. S1C). Flow cytometric analyses were performed within 4 h. For cytokine/chemokine analyses, plasma of PB samples collected during *in vivo* monitoring and supernatants of NK-DLI_IL-2 stim_ collected immediately prior to infusion, were stored at −80°C until analysis.

### Flow cytometric analysis for quantification of leukocyte subsets and cytotoxicity assay

Flow cytometric analyses were performed to determine (i) quality control of the administered NK-DLI, (ii) the specific influence of NK-DLI on the patient's immune system and (iii) the overall cellular immune reconstitution post SCT in all 16 patients. Monoclonal antibodies (mAB) conjugated with fluorescein-isothiocyanate (FITC), phycoerythrin (PE), phycoerythrin-Texas Red®tandem (ECD), phycoerythrin-cyanine-5 (PC-5) and phycoerythrin-cyanine-7 (PC-7) were used against following antigens (clones): CD3 (UCHT1) and (SK7)^#^, CD4 (13B8.2), CD4 (SFCI12T4D11), CD8 (SFCI21Thy2D3(T8)), CD14 (RMO52)^2^, CD14+CD16 (RMO522+3G8), CD16 (3G8), CD19 (J3-119), CD33 (D3HL60.251), CD45 (B3821F4A)^1^, CD45 (J.33), CD56 (N901) and (NCAM16.2)^1#^, CD62L (DREG56), CD69 (TP1.55.3)^1^, CD85k/ILT-3 (ZM3.8), CD123 (107D2), CD336/NKp44 (Z231), (^1^IgG2b, ^2^IgG2a, all other IgG1 isotypes) (^#^BD Biosciences, Heidelberg, Germany, all other Beckman Coulter, Marseille, France). For assessment of viability 7-Amino-Actinomycin D (7-AAD) was used. An automated lyse/no-wash procedure was used with a fixation step on a TQ-Prep™ Workstation (Beckman Coulter, Krefeld, Germany). Absolute lymphocyte subset counts were calculated via leukocyte counts measured by Coulter® Ac.T diff™Counter (Beckman Coulter, Krefeld, Germany). Measurements of myeloid DC, plasmacytoid DC and NK cells were carried out in a single-platform approach using Flow-Count™ fluorospheres (Beckman Coulter, Marseille, France) [17], [20]. On both, CD56^dim^CD16^+^ and CD56^bright^CD16^dim/−^ NK cells, surface expression of NKp44, CD69 activation and the lymph node homing molecule CD62L were investigated. NK cell cytotoxicity of NK-DLI_IL-2 stim_ and NK-DLI_unstim_ was tested against the MHC class I negative cell line K562 at the ratios 1∶1 and 10∶1 based on a 5-color flow cytometric single platform assay (Fig. S2A) [19]. Cytotoxicity was defined as the loss of viable target cells in relation to the mono-cultured control. All analyses were performed on a 4- or 5-color flow cytometer, respectively (EPICS® L™and FC500, Beckman Coulter, Krefeld, Germany) and data were further analyzed using CXP v2.2 software (Beckman Coulter, Krefeld, Germany).

### Cytokine and chemokine analysis

Cytokines and chemokines in the plasma of PB samples obtained during *in vivo* monitoring and supernatants of the 9–14 days stimulated NK-DLI_IL-2 stim_ were measured using BD™ Cytometric Bead Array (CBA) in combination with the BD FACSArray™ bioanalyzer (BD Biosciences, Heidelberg, Germany). The human Flex Set was used to detect the secretion of interleukin (IL) -1β, IL-2, IL-4, IL-6, IL-7, IL-8, IL-10, IL-12p70, IL-12/IL-23p40, IL-13, tumor necrosis factor-α (TNF-α), TNF-β, interferon-γ (IFN-γ) IFN-γ-inducible protein (IP-10), monocyte chemotactic protein-1 (MCP-1), macrophage inflammatory protein-α (MIP-1-α), MIP-1β, regulated on activation, normal T cell expressed and secreted (RANTES), Fas ligand (FasL), granulocyte colony-stimulating factor (G-CSF), granulocyte macrophage colony-stimulating factor GM-CSF. In short, 50 µl of the provided standards or plasma samples were mixed with capture beads specific for one cytokine and PE detection, and processed according manufacturer's instructions. The assay lower detection limit ranged from 0.2 to 14.7 pg/ml.

### KIR and HLA genotyping and chimerism analysis

Typing of KIR genes in PB of both, donors and patients was performed by PCR-sequence-specific primers and was used to detect the presence or absence of 19 KIR genes (2DL1-5B, 3DL1-3, 2DS1-5, 3DS1, 2DP1, 3DP1) as described previously [21]. HLA were typed using sequence-specific probes and sequence-based typing (SBT) analysis. KIR/HLA-ligand matching or mismatching in donor-recipient pairs was evaluated using the KIR receptor – HLA-ligand mismatch model (“missing KIR ligand model”). For quantitation of donor chimerism in the PB and bone marrow (BM), a semi-quantitative PCR assay based on the amplification of short tandem repeat (STR) markers was used [22].

### Statistical Analyses

Statistical analyses were performed using GraphPad Prism 5.03 (GraphPad Software, San Diego, USA). Biological data were compared by paired Student t test, Wilcoxon matched-pairs signed rank test and Mann-Whitney-test depending on paired or unpaired and Gaussian or non-Gaussian distribution of values. Differences were considered as significant for p<0.05, p<0.01, and p<0.001 indicated as *, **, and ***, respectively.

## Results

### Comparison of NK-DLI_unstim_ and NK-DLI_IL-2 stim_

In a clinical phase I/II study, 16 pediatric patients received either freshly isolated, unstimulated NK-DLIs (NK-DLI_unstim_; group I) or *ex vivo* IL-2 stimulated NK-DLIs (NK-DLI_IL-2 stim_; group II) from their respective donors, from one up to three times post haplo-SCT (Table 2). According to the study protocol, the median application date post SCT was day +2 (range 2–3), +42 (range 11–54) and +101 (range 70–126). After processing, the median purity of the CD56^+^CD3^−^ NK cell product was 95% (range 84.4–98.6). The majority of the concomitant cells were antigen presenting cells like monocytes and dendritic cells (mDC, pDC) as we could show previously [23]. The overall median yield during processing procedure was 54% (range 38.7–84.1). Patients of both groups received similar NK cell doses in the DLI: 14.6×10^6^/kg BW (range 6.0–45.1) and 13.1×10^6^/kg BW (range 3.2–38.3) for NK-DLI_IL-2 stim_ and NK-DLI_unstim_ with no differences between freshly and cryopreserved DLIs. Concomitant CD3^+^ T cells in the NK-DLI were higher in group II with a median of 34.6×10^3^/kg BW (range 7.7–98.3) compared to 4.3×10^3^/kg BW (range 0.4–53.4) in group I patients. Concerning fresh NK-DLIs this difference was not statistically significant, thus a statistical difference was seen in regard to all NK-DLI applications. Of note, of these overall CD3^+^ cells, in group II approximately two-thirds belonged to the CD56^+^CD3^+^ NK-like T cells and one-third to CD56^−^CD3^+^ T cells, only. Median NK cell cytotoxicity against MHC class I negative K562 cells in the effector∶target ratios 1∶1 and 10∶1 was 67% and 88% for NK-DLI_IL-2 stim_ compared to 30% and 75% for NK-DLI_unstim_ (Fig. S2A). The improved cytotoxicity of NK-DLI_IL-2 stim_ was related to a high up-regulation of the activating receptors NKp30, NKp44, NKp46 and NKG2D. The CD69 activation marker expression was strongly enhanced as well, whereas the lymph node homing molecule CD62L was significantly down-regulated upon IL-2 stimulation as we showed previously [16]. Freshly administered NK-DLI_unstim_ showed a high viability (median 93%) compared to NK-DLI_IL-2 stim_ which showed a decrease in vital NK cell count to 30–70% during the first three days, followed by a period of enhanced growth and increasing viability during 9–14 days of IL-2 expansion [16], [18].

The applied CD34^+^ stem cell dose in the graft was similar in both groups with a 6.2 times higher CD3^+^ T cell count in grafts of group II compared to group I patients (13.5×10^3^/kg BW vs. 84.5×10^3^/kg BW, difference not significant). This was due to a change in graft purification from CD34-selection to CD3/CD19-depletion (Table 1). Therefore, patients transplanted with CD3/CD19 depleted grafts received mycophenolate mofetil (MMF) as an immunosuppressive therapy to avoid severe GvHD and graft rejection. Because of a possible negative impact of MMF on NK cell functionality, the early time point of NK-DLI at day +3 post SCT has been omitted for these patients. To validate if the differing graft purifications in group I have any influence of our presented results, we exemplarily excluded the four patients receiving CD34 selected grafts from our analyses, whereof only two fresh NK-DLI_unstim_ were applied (data not shown). Since we did not see any differences in our results and it confirmed the overall conclusion, we decided to remain with the total patient group.

NK-DLI was well tolerated in both groups, besides transient fever and chills for 24 h in group II patients receiving NK-DLI_IL-2 stim_. In two patients (No. 9 and 13) steroids were temporarily administered during NK-DLI because of more adverse effects (vomiting and blood pressure fluctuation). Patients of group II did not develop GvHD>grade II in response to NK-DLI_IL-2 stim_, independent of the amount of stimulated T cells. In group I, one patient (No. 1) treated with NK-DLI_unstim_ containing >50.000×10^3^/kg BW CD3^+^ cells and the patient with the highest amount of residual T cells in the graft (No. 5) developed GvHD>grade II. Following the full myeloablative conditioning regime, patients were in aplasia until engraftment was seen in both patients' subgroups, excluding patient No. 2 and No. 16, on days 7, 12 and 14 for platelets (>50.000/µl), leukocytes (>1000/µl) and neutrophils (>1000/µl), respectively.

Therefore, data of all day +3 NK-DLI_unstim_ applications were not evaluable, since to that early time point post SCT, patients were still in aplasia (median cell counts in PB at day +3: leukocytes 120/µl, lymphocyte 0/µl, NK cells 0/µl, monocytes 0/µl). Because infusion of NK-DLI_unstim_ at day +3 did not lead to any changes, analyses were not included in any of the following figures. Specific immune reconstitution of CD56^+^CD3^−^ NK cells post haplo-SCT was very similar in both groups (Fig. S2B). Chimerism analyses of PB and bone marrow beginning between day +11 and +15 showed complete donor chimerism in 14/16 patients to that early time point post SCT. Complete chimerism retained in 14/16 patients at day +40 and in 14/15 at day +100 post haplo-SCT (Table 1). Also subtype chimerism analyses of CD56^+^ NK cells were performed in 5/16 patients which reflected results of total PB and BM chimerism (data not shown). The only patient that did not reach complete chimerism at day +40 (No. 2) rejected the stem cell graft and underwent a second SCT. Further, in patient No. 16 no chimerism analyses could be performed because of failure of immune reconstitution and early death at day +27.

Overall, 7/9 patients receiving NK-DLI_IL-2 stim_ and 5/9 receiving NK-DLI_unstim_ have not been in remission (NR) or in partial remission (PR) at the time point of SCT, while all patients in remission were ≥second complete remission (CR2). Furthermore, 44% of these high risk patients are alive with regard to both NK-DLI_IL-2 stim_ and NK-DLI_unstim_ groups with a mean follow up of 20 and 45 months, respectively. KIR mismatch was seen in 6/9 patients of group I and 8/9 in group II in GvL/T direction and in 6/9 in both groups in HvG direction (Table 2). Nevertheless, in this heterogeneous patient cohort, no clear influence on survival, GvHD and rejection with regard to KIR mismatch could be seen.

Of note, effects of NK-DLI_IL-2 stim_ application on PB leukocytes in patient No. 16 were not evaluable because of failure of immune reconstitution (Table 1). Further, measurements of the 2^nd^ NK-DLI_IL-2 stim_ application of patient No. 14 (d +98, cryopreserved) were excluded, since the DLI has been washed prior to administration because the patient had shown severe reaction to DMSO previously.

### NK-DLI_IL-2 stim_ but not NK-DLI_unstim_ led to a significant decrease of CD56^+^CD3^−^ NK cells in patient's PB

Monitoring of the leucocyte subsets in patient's PB before and 10 min, 1 h, 4 h and 24 h after NK-DLI was performed to receive first insights of the host immune reaction on donor NK cell immunotherapy (Fig. S1C). We focused our main interest on fresh NK-DLI applications, because the cryopreservation process (cell centrifugation, concentration in a smaller volume and dilution in DMSO) results in cell count and cytokine/chemokine reduction and could lead to an impairment of NK cell and cytokine functionality.

**Figure 1:**
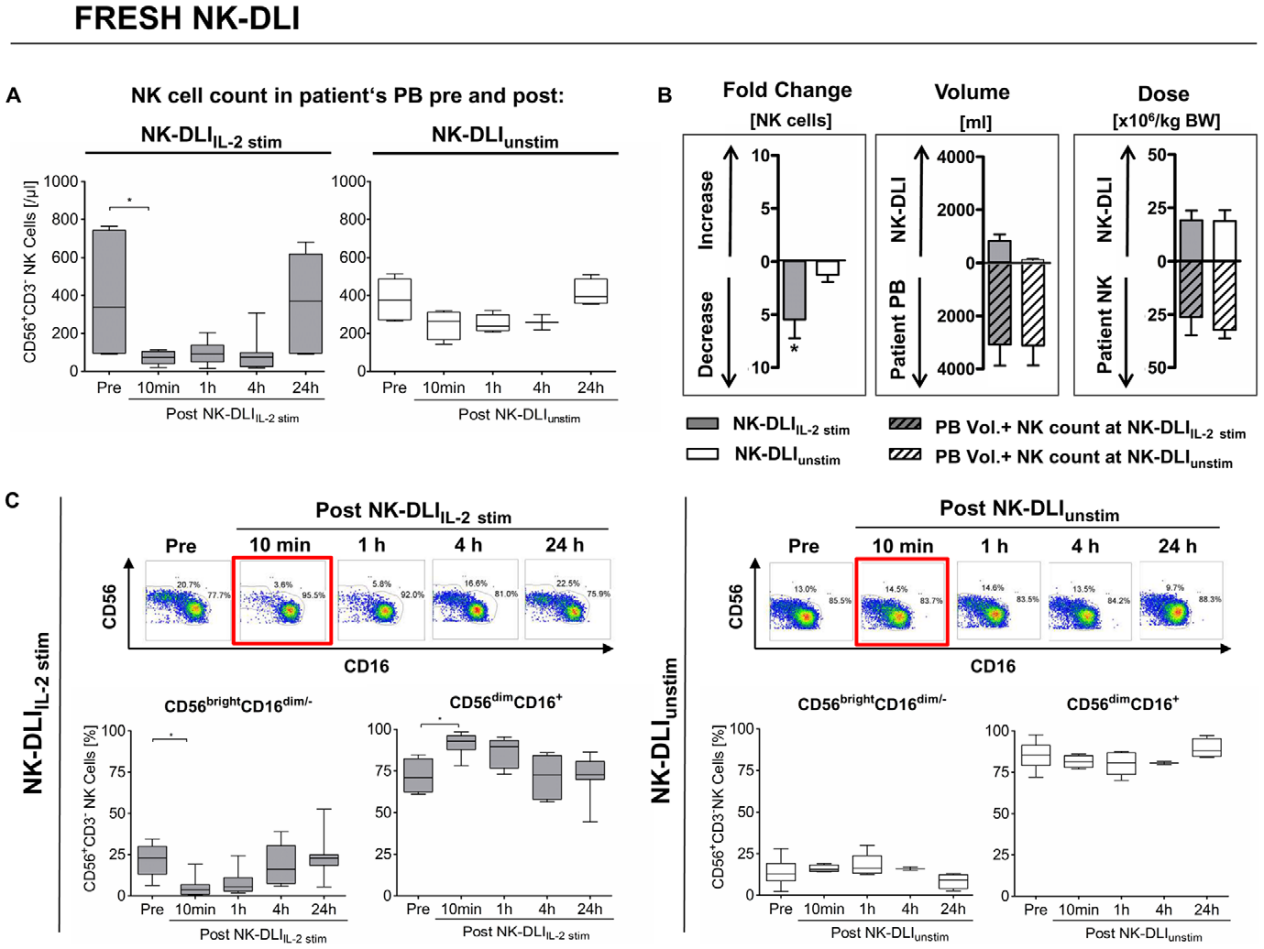
NK-DLI_IL-2 stim_ but not NK-DLI_unstim_ led to a considerable disappearance of NK cells from PB. A) Absolute number of NK cells in the PB of the patients was significantly reduced 10 min post NK-DLI_IL-2 stim_ (grey) in contrast to NK-DLI_unstim_ (white), which showed minimal influence only. 24 h after NK-DLI_IL-2 stim_ absolute number of NK cells recovered to the level before DLI. Only freshly applied NK-DLIs infused around day +40 post SCT are shown and were used for statistical calculations (n = 7 NK-DLI_IL-2 stim_, n = 6 NK-DLI_unstim_). Box and whiskers plots show minimum, lower quartile, median, upper quartile and maximum of all measured data. For the 4 h level post NK-DLI_unstim_ two values were available, only. p<0.05 indicated as *. B) Mean and SEM of all freshly applied NK-DLI_IL-2 stim_ (grey) and NK-DLI_unstim_ (white) applied around day +40 post SCT. Striped bars indicate estimated patient's PB volume and peripheral NK cell count at the time point of NK-DLI infusion. Left graph shows a mean 5.5-fold reduction of absolute NK cell count in the PB as early as 10 min post NK-DLI_IL-2 stim_ compared to a 1.2-fold decrease for NK-DLI_unstim_. Middle graph shows relation of NK-DLI volume to total blood volume of patients (NK-DLI_IL-2 stim_ volume: 825 ml±249, compared to PB volume: 3060 ml±789; NK-DLI_unstim_ volume: 128 ml±19, compared to PB volume 3093 ml±749). DLI volume did not artificially lead to the reduced absolute NK cell count, since NK-DLI_IL-2 stim_ volumes made up maximally around ¼ of patient's PB volume. Right graph shows mean NK-DLI cell dose ×10^6^/kg BW (NK-DLI_IL-2 stim_ 19.2±4.5; NK-DLI_unstim_ 18.8±5.1) in relation to patient's PB NK cells newly reconstituted post haplo-SCT around d +40 prior to NK-DLI (NK-DLI_IL-2 stim_ 26.0±8.5; NK-DLI_unstim_ 31.9±4.0). Applied NK dose compromised about 80% of PB NK cells, illustrating the high dose of administered NK-DLI. C) Density plots (CD56 vs. CD16) and box and whiskers plots show a significant change in the distribution of the cytotoxic CD56^dim^CD16^+^ and immune regulatory CD56^bright^CD16^dim/−^ NK cell subsets. This was due to an absolute reduction of the CD56^bright^CD16^dim/−^ NK cell subpopulation in the PB 10 min after freshly applied NK-DLI_IL-2 stim_ applications infused around d +40 (left; n = 7). This could not be shown after NK-DLI_unstim_ (right; n = 6). Plots are gated on CD56^+^CD3^−^ NK cells. For the 4 h level post NK-DLI_unstim_ 2 values were available, only. p<0.05 indicated as *.

All NK cell infusions were associated with a reduction in circulating NK cells within 10 min of infusion, but this was significantly greater in patients receiving NK-DLI_IL-2 stim_ (Fig. 1A fresh NK-DLIs). After a 5.5-fold decrease in absolute NK cell counts 10 min post infusion of freshly applied NK-DLI_IL-2 stim_ in contrast to NK-DLI_unstim_ (1.2-fold) (Fig. 1B, left), NK cell counts recovered within 24 h to prior values. DLI volume did not artificially lead to the reduced absolute NK cell count, since NK-DLI_IL-2 stim_ volumes made up around ¼ of patient's PB volume, respectively (Fig. 1B, middle). In addition, in both subgroups the ratio of the administered NK-DLI dose compared to the patient's NK cells in the PB newly reconstituted post haplo-SCT was very similar (Fig. 1B, right). Importantly, the decrease in CD56^+^CD3^−^NK cells was not due to a down-regulation or loss of CD56 expression (Fig. S3B).

Effects of total NK-DLI_IL-2 stim_ applications (fresh and cryopreserved) were similar, thus NK cell disappearing was delayed, compared to the fast diminishing after fresh infusions (compare Fig. 1A and Fig. S4 total NK-DLIs).

Moreover, infusion of NK-DLI_IL-2 stim_ led to a change in the proportion of CD56^bright^CD16^dim/−^ and CD56^dim^CD16^+^ NK cell subpopulations which was due to a more pronounced loss of CD56^bright^CD16^dim/−^ NK cells (Fig. 1C fresh NK-DLIs). Similar effects were seen after total NK-DLI_IL-2 stim_ applications (Fig. S4 total NK-DLIs). Patients receiving NK-DLI_unstim_ showed no change in the proportion of NK cell subpopulations.

Of note, NK cells with the characteristics of the *ex vivo* IL-2 activated phenotype (CD56^bright^CD16^+/−^CD69^+^NCR^high^CD62L^−^) could not be detected in the PB at any time during *in vivo* monitoring, thus not returning post 24 h (Fig. S3A+B). In addition, CD62L^+^ expressing PB NK cells declined 10 min post NK-DLI_IL-2 stim_ in the same manner like total NK cells, also recovering within 24 h (Fig. S3B+C). Therefore, reduction in total NK cell count post NK-DLI_IL-2 stim_ was due to both, a rapid decrease in transferred and peripheral blood NK cells.

### Loss of antigen presenting cells (APCs) in the PB after infusion of NK-DLI_IL-2 stim_ but not after NK-DLI_unstim_

**Figure 2:**
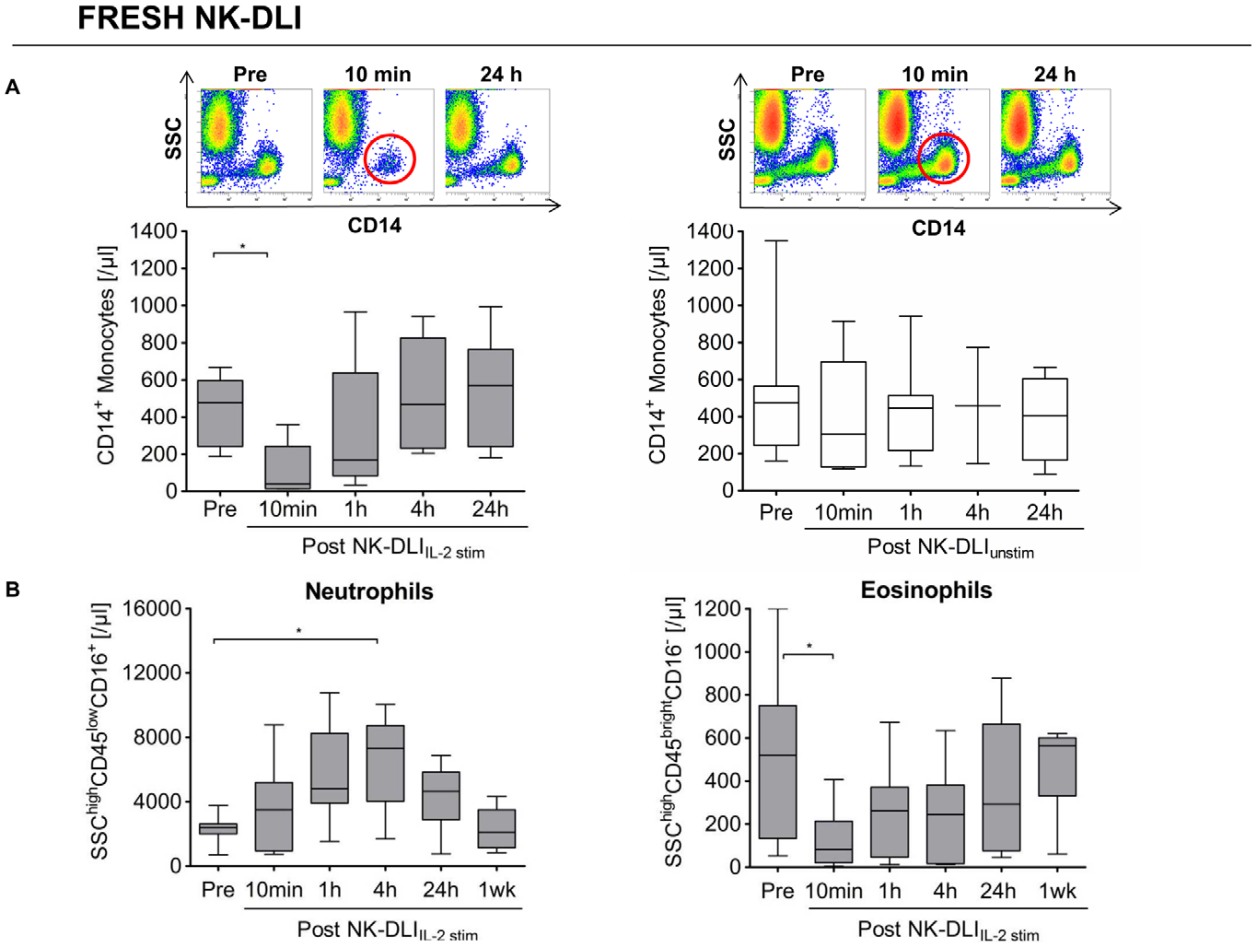
Influence of NK-DLIs on monocyte and granulocyte distribution in patient's PB. A) Significant reduction of absolute CD14^+^ monocyte count could be demonstrated in patient's PB 10 min after application of fresh NK-DLI_IL-2 stim_ (n = 7), while recovering to normal values within the next 24 h. This was not seen after NK-DLI_unstim_ (n = 6). Density plots show side scatter (SSC) vs. CD14 gated on CD45^+^ leukocytes. For the 4 h level post NK-DLI_unstim_ 2 values were available, only. p<0.05 indicated as *. B) We observed a distinct influence of NK-DLI_IL-2 stim_ on neutrophil and eosinophil granulocyte count. A massive increase of neutrophils (SSC^high^CD45^low^CD16^+^) with its peak at 4 h was combined with a significant reduction of eosinophil granulocytes (SSC^high^CD45^bright^CD16^−^) in the PB after fresh NK-DLI_IL-2 stim_ (n = 7). This was not observed after unstimulated NK cell applications (data not shown). p<0.05 indicated as *.

Shortly after infusion of NK-DLI_IL-2 stim_ there was a significant loss of CD14^+^ monocytes in the PB of all patients (median 15-fold reduction). After disappearing almost completely, monocyte counts recovered within 4 to 24 h to normal values like the ones prior to infusion. This was the case following fresh NK-DLI_IL-2 stim_ as shown in Fig. 2A, as well as after all NK-DLI_IL-2 stim_ applications in total (Fig. S4 total NK-DLIs). In contrast, monocyte counts following NK-DLI_unstim_ remained unaffected. Furthermore, total CD45^+^CD14^−^CD16^−^HLA-DR^+^CD85k^+^CD33^+^ myeloid dendritic cell (mDC) and CD45^+^CD14^−^CD16^−^HLA-DR^+^CD85k^+^CD123^+^ plasmacytoid dendritic cell (pDC) count also significantly decreased directly after NK-DLI_IL-2 stim_, recovering subsequently to normal values within 24 h to 1 week (Fig. S4). Again, these effects could not be observed after infusion of NK-DLI_unstim_. Measurements of DCs were carried out in a single-platform approach verifying reliable data even in low cell counts.

### Distinct influence of NK-DLI_IL-2 stim_ and NK-DLI_unstim_ on granulocytes, and T and B cells in the PB

Following all applications of NK-DLI_IL-2 stim_ a significant loss of eosinophil granulocytes (SSC^high^CD45^bright^CD16^−^) combined with a massive increase in absolute white blood cell (WBC) count was observed (average 4-fold). This was caused by an intensive gain of the main CD45^+^ leukocyte proportion of neutrophil granulocytes (SSC^high^CD45^low^CD16^+^) leading to its peak after 4 h and then returning to normal values prior to infusion (Fig. 2B fresh NK-DLIs). Similar effects were seen after total NK-DLI_IL-2 stim_ applications (Fig. S4 total NK-DLIs). In contrast, the distinctly increased absolute WBC count in the PB of the monitored patients could not be noted after NK-DLI_unstim_ applications (data not shown).

According to a delayed immune reconstitution of T and B cells in the patients' PB post haplo-SCT compared to an early NK cell recovery, CD3^+^ T cell and CD19^+^ B cell counts at the time point of NK-DLI at day +40 were relatively low (median 50/µl CD3^+^, and 58/µl CD19^+^). NK-DLI_IL-2stim_ led to a very slight decrease in absolute T cell count within 4 h post NK-DLI_IL-2 stim_ while B cell count was not affected (data not shown).

### Only after NK-DLI_IL-2 stim_ a significant increase in cytokine/chemokine concentration in patients' PB was demonstrated, thus varying from levels from the *ex vivo* engineered DLI

**Figure 3:**
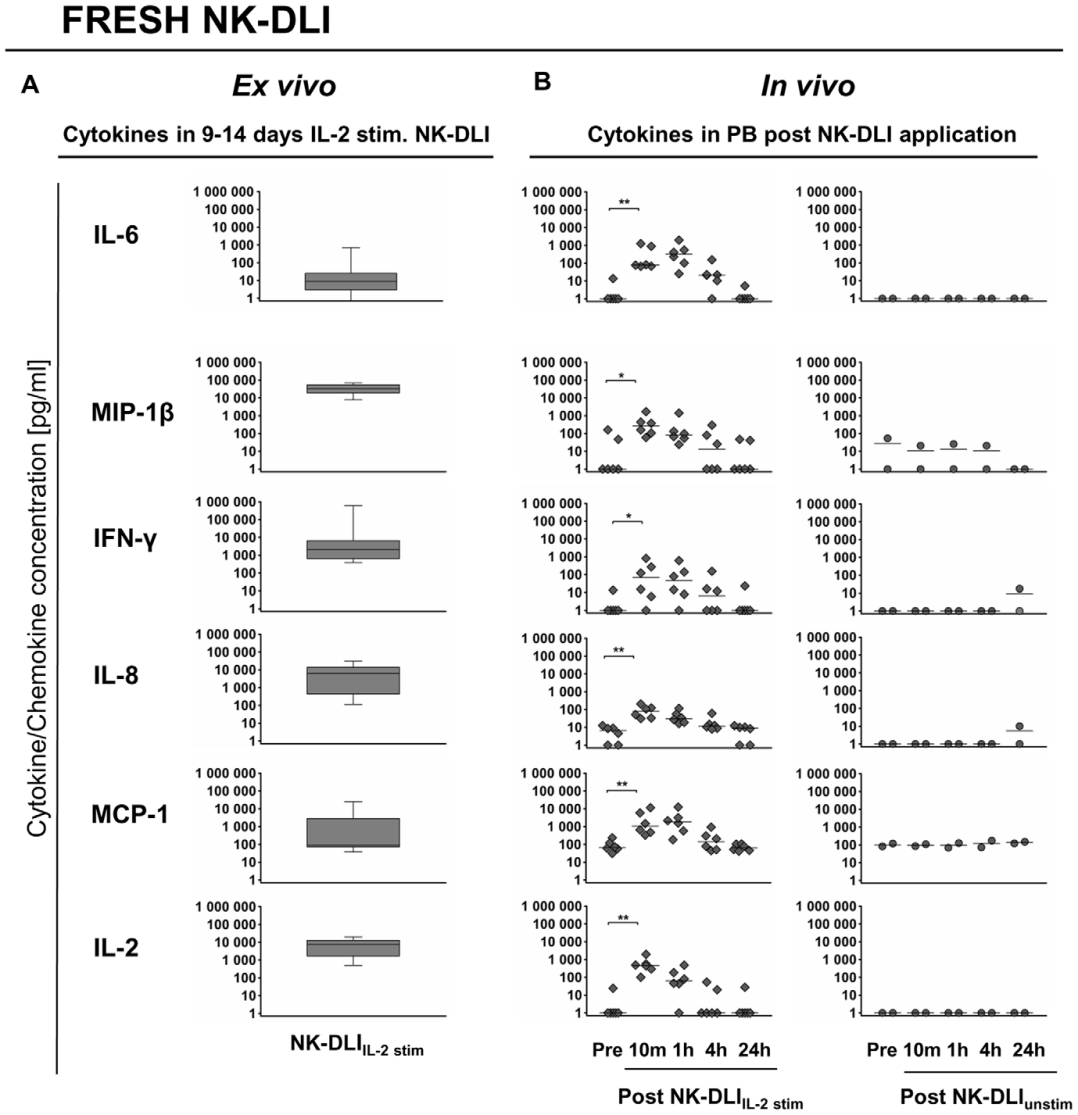
*In vivo* monitoring of patient's cytokine/chemokine plasma levels following NK-DLI. A) Box and whiskers plots show the respective cytokine/chemokine concentration present in the NK-DLI_IL-2 stim_ product (n = 12) immediately prior to infusion. The nine to 14 days *ex vivo* IL-2 stimulation of highly purified donor NK cells led to the secretion of high amounts of various chemokines i.e. IL-8 and MIP-1β, as well as the pro-inflammatory cytokine IFN-γ. Whereas, the median concentration of the pro-inflammatory cytokine IL-6 was <10 pg/ml. The indicated IL-2 concentration was due to addition during expansion procedure (see Material and Methods). Y-axis shows cytokine/chemokine concentration, range 1–1.000.000 pg/ml. B) Cytokine analyses of cryopreserved plasma samples collected before (pre) and 10 min, 1 h, 4 h and 24 h after fresh NK-DLI_IL-2 stim_ (⧫, n = 6). Significant increases of i*n vivo* cytokine/chemokine concentration of IL-2, IL-6, IL-8, IFN-γ, MCP-1 and MIP-1β in patient's plasma following NK-DLI_IL-2 stim_ were observed. Levels peaked after 10 min to 1 h post infusion, remained enhanced over a period of 4 h and returned to base level within the next 24 h. In contrast, no increase in cytokine/chemokine PB concentration following NK-DLI_unstim_ was seen (•, n = 2). p<0.05 and <0.01 indicated as * and **.

*Ex vivo* IL-2 stimulation of highly purified donor NK cells led to the secretion of high amounts of various chemokines e.g. IL-8, MIP-1β, MCP-1, IP-10 and RANTES which play crucial roles in leukocyte activation and chemo-attraction, as well as the inflammatory cytokine IFN-γ (Fig. 3A). In contrast, the median concentration of the pro-inflammatory cytokine IL-6 was below 10 pg/ml. The indicated IL-2 concentration was due to the supplementary addition of 1000 U/ml IL-2 during expansion procedure. Since NK-DLIs have not been washed prior to infusion according to the study protocol, to avoid loss of NK cells, the total amount of the indicated cytokines/chemokines was applied during fresh NK-DLI_IL-2 stim_.

*In vivo* analyses of cytokine/chemokine concentration in patient's PB post NK-DLI_IL-2 stim_ revealed significant increases in plasma levels up to 4 h following infusion. The pro-inflammatory cytokine IL-6 and IFN-γ, as well as the chemokine IL-8, MCP-1 and MIP-1β plasma concentrations peaked within 10 min to 1 h post NK-DLI_IL-2 stim_ while recovering to their base level within the next 24 h (Fig. 3B fresh NK-DLIs). In addition, high plasma levels of the chemokines IP-10 and RANTES, acting on both leukocyte activation and attraction, were detected but not varying (data not shown). Similar results post infusions of cryopreserved NK-DLI_IL-2 stim_ were obtained, but to a slighter extent, which was probably due to a reduction in cytokine/chemokine concentration during the cryopreservation process. Nevertheless, analyses of all NK-DLI_IL-2 stim_ in total showed identical results and did not change the overall conclusion (Fig. S4 total NK-DLIs). In comparison, no increase in plasma concentration of the indicated cytokines/chemokines following NK-DLI_unstim_ was detected (Fig. 3B). Besides, TNF-α, TNF-β, GM-CSF, MIP-1α, FasL, IL-13 were secreted during *ex vivo* IL-2 NK cell expansion, but either they could not be detected in patient's PB, or NK-DLI_IL-2 stim_ did not lead to any changes in present plasma concentrations (data not shown). IL-1β, IL-4, IL-7, IL-10, IL-12p70, IL12/23 and G-CSF were analyzed as well, but were not detected in NK-DLI and PB.

When focusing on one hand on the *ex vivo* cytokine/chemokine levels in the NK-DLI_IL-2 stim_ products and on the other hand on patients' PB levels measured directly after infusion, marked variations occurred. Being aware that besides other factors i.e. dilution effects need to be considered, IL-2, IL-8, IFN-γ and especially MIP-1β were found in lower concentrations in the PB than in the NK-DLI_IL-2 stim_ (Fig. 3A+B).

## Discussion

Here we present first interesting insights from our clinical NK cell phase I/II study using allogeneic NK-DLI_unstim_ compared to NK-DLI_IL-2 stim_ in pediatric patients suffering from high risk malignancies. Although, we, among others, have shown that the infusion of unstimulated as well as previously *ex vivo* IL-2 stimulated allogeneic NK cells post haplo-SCT is well tolerated without inducing severe GvHD>grade II [2], [12], [14], [16], possible risks or disadvantages need to be critically discussed. As it stands, literature is scarce about the fate and behavior of adoptively transferred allogeneic NK cells in humans and about the potential distinct influences of unstimulated NK cells in contrast to previously *ex vivo* activated NK cells on patient's adaptive and innate immune system.

Several studies in animals have addressed the question concerning the capability to traffic to specific tissues, the regulation of homing, and the survival of adoptively transferred cells *in vivo*. NK cell trafficking to spleen, lymph nodes, lung, liver, gastrointestinal tissue and tumor side with a survival up to four weeks following transfer was observed by a bioluminescence-based strategy, which correlated with an observed anti-tumor effect [24]–[26]. However, to date only one small clinical trial in humans was performed, where three adult patients with renal cell carcinoma received stimulated allogeneic NK cells labeled with the radioactive substance Indium-111 oxine [27]. After an initial accumulation in the lungs, NK cells redistributed to liver, spleen and bone marrow as well as in two of four metastases in lung and liver. Unfortunately, it has been reported as well that Indium labeling significantly affects the cellular integrity [28]. Even though it is still of particular interest if adoptively transferred NK cells in humans actually reach their side of action, clinical trials using NK cell labeling with potentially harmful substances will not obtain approval in the treatment of pediatric malignancies. Therefore, approaches using more noninvasive strategies have to be considered. Our investigation is based on a comprehensive *in vivo* cytokine/chemokine monitoring and on flow cytometric analyses of quantification, constitution and distribution of various PB leukocyte subsets before and after NK-DLI application.

In our study we have reported markedly diverse effects between NK-DLI_unstim_ and NK-DLI_IL-2 stim_. Shortly after infusion of NK-DLI_IL-2 stim_ only, a rapid almost complete loss of cells dominantly from the innate immune system from patient's PB circulation appeared which was accompanied by significant increases in plasma concentration of various cytokines and chemokines. Whereas neutrophil granulocytes markedly increased within 4 h post NK-DLI_IL-2 stim_, monocytes, dendritic cells, eosinophils and especially NK cells massively decreased as early as 10 min post infusion, while recovering within the next 24 h.

Moreover, when analyzing NK cells more into detail, we were able to clearly discriminate between adoptively transferred and patients' PB NK cells by a distinct CD69, NCR and CD62L expression. We have shown previously that *ex vivo* IL-2 stimulation leads to a predominantly CD56^bright^CD16^+/−^ phenotype with a strongly enhanced expression of the activation marker CD69, while CD62L becomes down-regulated. Further, surface receptors involved in NK cell cytotoxicity become highly up-regulated. While only one-third of unstimulated NK cells, a median of 95% of IL-2 stimulated NK cells show expression of NCRs. In detail, NKp44, NKp30, NKp46 and NKG2D expression significantly increased 33-fold, 12-fold, 3-fold and 4-fold, respectively [16]. Furthermore, the IL-2 stimulation led to a consistent increase in NK cell killing activity against a neuroblastoma cell line [16] and the leukemic cell line K562 (Fig. S2A). In addition, Penack *et al.* showed that the CD16^−^ NK cell subset is responsible for anti-tumor responses [29].

NK cells with the characteristics of the *ex vivo* IL-2 stimulated phenotype (CD56^bright^CD16^+/−^CD69^+^NCR^high^CD62L^−^) were not detected in patients' PB at any time point during *in vivo* monitoring. Furthermore, we could clearly show that the significant reduction of CD56^+^CD3^−^ NK cells from blood circulation following NK-DLI_IL-2 stim_ was due to both, a decrease in patients' own PB CD62L^+^ NK cells as well as a rapid diminishing of the transferred, stimulated NK cells from the NK-DLI with the CD62L^−^ phenotype.

In contrast, PB cell subpopulations remained constant after NK-DLI_unstim_. This effect was not due to NK-DLI dose, a PB dilution effect after infusion, application date or host's NK cell immune reconstitution. All these variables were very similar in both, patients receiving NK-DLI_unstim_ and those receiving NK-DLI_IL-2 stim_. The only difference was the IL-2 for generation of NK-DLI_IL-2 stim_ and the high amount of cytokines and chemokines such as IFN-γ, IL-8, MCP-1, IP-10, RANTES, MIP-1β secreted in the course of *ex vivo* expansion. Those factors were only transfused to patients treated with NK-DLI_IL-2 stim_.

In accordance with our results, early studies have reported a rapid diminishing of various types of PB lymphoid cells, especially NK cells, 15 min after *in vivo* bolus single cytokine administration of very high doses of recombinant IL-2 (up to 1×10^6^ U/kg BW). Similar to our study, cells also recovered within the next 24 h. Furthermore, IL-2 was rapidly cleared from the plasma with a half-life of 6.9 min [30], [31]. It has been suggested that the IL-2 induced disappearance of NK cells may be related to a massive adhesion to the activated endothelium [31], [32]. Our observed effects cannot be attributed to one single cytokine/chemokine but to the whole cytokine “cocktail” applied with the NK-DLI_IL-2 stim_ product, but the IL-2 dose applied by our NK-DLI study was extremely lower (∼2×10^4^ U/kg BW) in comparison to the discussed data by Lotze *et al.* (∼1×10^6^ U/kg BW). Apparently, much lower concentrations of IL-2 but in combination with our indicated cytokines/chemokines administered by NK-DLI_IL-2 stim_ led to a comparable effect to high dose single IL-2 application with regard to PB leukocyte diminishing.

Measuring cytokine/chemokine production is an integral part of measuring immune response during immunotherapy. Because cytokines act in networks and have overlapping functions, monitoring of a single cytokine may be of limited use [33]. Following NK-DLI_IL-2 stim_ we have shown significant increases in plasma concentration of several chemotactic and inflammatory cytokines and chemokines which remained enhanced up to 4 h post DLI (Fig. 3B). The majority of the analyzed increases were probably induced by the infusion of high amounts of *ex vivo* generated cytokines/chemokines in the NK-DLI_IL-2 stim_. We assume that these changes in the natural cytokine milieu of the PB led to the observed cell migration processes. The massive increase of blood neutrophils, which represent the major early cell type to invade inflammatory foci, is likely mediated by the transfer of high amounts of IL-8 that were produced in the course of *ex vivo* NK cell stimulation. Notably, IL-8 has been described to be the major chemo-attractant for neutrophil granulocytes. Neutrophils are described to be potent producers of various cytokines (i.e. IL-6, IL-8, IP-10, MIP-1α/β) which may be in relation to the prolonged enhanced cytokine/chemokine levels 4 h post NK-DLI_IL-2 stim_ application [34], [35].

Furthermore, the disappearance of various leukocyte subsets occurring only after NK-DLI_IL-2 stim_ may be mediated by two alternative or complementary mechanisms: (i) adherence to the activated endothelium induced by high amounts of co-infused cytokines/chemokines, (ii) leukocyte migration from the PB into the extravascular compartment.

Although normal endothelial cells exhibit low affinity for circulating lymphocytes, the high amount of the cytokines and chemokines present in the PB (i.e. IFN-γ, MIP-1β, IL-8), similar to those released in the course of inflammation and other immune reactions, leads to endothelial activation associated with an increased expression of surface antigens which interact with all leukocytes [31]. This might result in endothelial adherence and therefore diminishing of leukocytes from blood circulation. Further, it is known that soluble cytokines and chemokines bind endothelial molecules including glycosaminoglycans (GAGs) and the Duffy antigen/receptor for chemokines (DARC), which are involved in the trans-endothelial transport of several chemokines, i.e. MIP-1β, IL-8, RANTES, MCP-1 and IP-10 [36]–[38]. Chemokines bound at the luminal endothelial cell surface could provide a trans-cellular chemotactic gradient guiding leukocyte extravasation [39]. Therefore, we assume that following the firm attachment to the activated endothelium, the cells migrate across the endothelium barrier into the tissue, actually leaving PB blood circulation. Although 7-AAD analyses revealed no increase in dead cells over the whole period of *in vivo* monitoring (data not shown), a cell reduction in PB circulation due to cell death cannot be excluded completely.

In addition to the discussed trans-endothelial transport of cytokines/chemokines, cytokine stability in circulation, renal clearance as well as dilution effects during infusion must be regarded as parameters that have likely contributed to the described discrepancy between high *ex vivo* levels in the NK-DLI and much lower *in vivo* PB levels. In our study we found a significant reduction of the immune regulatory CD56^bright^CD16^dim/−^ NK cell subpopulations 10 min after NK-DLI_IL-2 stim_. An explanation for the overall higher susceptibility of the CD56^bright^CD16^dim/−^ NK cell subpopulation might be the high expression of various chemokine receptors i.e. the MIP-1β corresponding CCR5 receptor on the cell surface of the CD56^bright^CD16^dim/−^ subpopulation, only [40].

Finally, IL-6 was the only cytokine which was secreted much higher in the PB of our patients compared to those during NK cell expansion, leading us to suspect a secondary production of the patient's body in response to NK-DLI_IL-2 stim_. We speculate that this could be due to both, the secretion of endothelial cells in reaction to the changing cytokine milieu, neutrophil granulocytes and monocytes that transiently adhere to the endothelial surface. In addition, the increase of IL-6 in patients' blood plasma as a response to NK-DLI_IL-2 stim_ correlated with our clinical observations of transient fever and chills; therefore serving as a surrogate marker of the biological activity of the *ex vivo* secreted and co-infused cytokines and chemokines.

Till now, very little is known about the effects of NK cell administration post SCT. These concomitant results to a clinical immunotherapy study provide first insights on the distinct influence of unstimulated vs. *ex vivo* IL-2 stimulated NK cell infusions. Nevertheless, we are fully aware that dissimilarities in the study design and the heterogeneous small patient cohort may have a potential effect on the results and that further studies have to verify the discussed data.

Moreover, an open issue remains the clinical benefit of NK-DLI_IL-2 stim_ compared to NK-DLI_unstim_ applications. Due to our heterogeneous patient cohort regarding different high risk diseases, with multiple and advanced relapses, mostly not in remission (NR), a clear evidence cannot be made. Anyhow, in the present study we could show a superior cytotoxicity of *ex vivo* IL-2 stimulated compared to unstimulated NK cells against the MHC-I negative cell line K562 and against a neuroblastoma (NB) cell line as well [16]. Cautiously it has to be noted, that 78% of the high risk group II patients treated with NK-DLI_IL-2 stim_ has not been in remission during haplo-SCT, but reached a survival of 44%. In addition in this group, two out of four patients suffering from high risk NB stadium IV with a very poor prognosis are still alive >2 years post NK-DLI_IL-2 stim_, which seems promising and is in accordance with the enhanced lytic activity of IL-2 stimulated NK cells compared to NK-DLI_unstim_ against NB [16].

Conclusively, we were able to show that the adoptive transfer of NK-DLI_IL-2 stim_ results in massive cell migrating processes under the influence of various *ex vivo* and most likely also *in vivo* secreted cytokines and chemokines. Since IL-2 activation leads to an improved cytotoxic capacity of the adoptively transferred NK cells, the co-transfused cytokine milieu may promote NK cell trafficking as well as an enhanced efficacy of NK cell immunotherapy.

## Supporting Information

Figure S1 **Study designs of the clinical phase I/II NK-DLI and our concomitant** ***in vivo*** **monitoring analyses.** A) In a phase I/II clinical feasibility study starting in the year 2003, haploidentical donor NK cells were isolated from unstimulated leukapheresis and purified by a two-step CD3-depletion/CD56-selection procedure. For haplo-SCT (d 0), peripheral blood stem cells (PBSC) were purified immunomagnetically either by CD34-selection or CD3/CD19-depletion. For NK cell collection, leukapheresis was performed at day −10 prior and +40 post SCT. At day +40, NK-DLI_unstim_ was applied freshly, directly at the end of the purification process, while the processed NK-DLI_unstim_ from day −10 was split and cryopreserved for the NK cell application on day +3 and +100. B) In an amendment of the study starting in the year 2005 two leukapheresis products collected on day +29 and +30 post SCT were pooled for the CD3-depletion/CD56-selection NK cell purification process. Following purification, NK cells were further *ex vivo* expanded and activated using 1000 U/ml IL-2 for 10 (9 to 14) days obeying GMP. After *ex vivo* stimulation, the NK cell product was split up, while one half was infused freshly at day +40 and the other was cryopreserved and applied at day +100 post SCT. For haplo-SCT (d 0), peripheral blood stem cells (PBSC) were purified immunomagnetically by CD3/CD19-depletion. C) For our concomitant *in vivo* monitoring study during NK-DLI, PB samples were collected at the day of application before (pre), 10 min, 1 h, 4 h and 24 h after the end of NK-DLI application.
(TIF)

Figure S2 A) **NK cell cytotoxicity of NK-DLI_unstim_ vs. NK-DLI_IL-2 stim_.** Cytotoxic activity of donor NK cells against K562 was significantly enhanced by IL-2 stimulation. The killing activity against the MHC class I negative leukemic cell line K562 of IL-2 stimulated NK-DLIs (grey, n = 9) was significantly greater compared to unstimulated NK cells (white, n = 9) at both effector∶target ratios 1∶1 and 10∶1. NK cell cytotoxicity of freshly isolated unstimulated or IL-2 stimulated products was tested previously to application to the patients and/or before cryopreservation. Cytotoxicity was analyzed based on a 5-color flow cytometric single platform assay [19] and defined as the loss of viable target cells in relation to the mono-cultured control. p<0.01 and <0.001 indicated as ** and ***. B) ***In vivo*** **NK cell immune reconstitution post haplo-SCT.** Similar NK cell immune reconstitution post haplo-SCT in both patients' groups receiving NK-DLI_IL-2 stim_ and NK-DLI_unstim_ respectively. Very similar NK cell immune reconstitution was seen in both patient subgroups NK-DLI_unstim_ (□, n = 7) and NK-DLI_IL-2 stim_ (▴, n = 6). Immune reconstitution of all patients was monitored regularly; within the first three months post SCT weekly, from month four to six twice a month, followed by a period of monthly analyses. Shown are all measurements and median performed in each interval which were similar in both groups.
(TIF)

Figure S3 ***In vivo*** **NK cell phenotype in patients PB differs from that of** ***ex vivo*** **expanded NK-DLI_IL-2 stim_.** A) Upon 9–14 days of IL-2 stimulation, CD56, NKp44 and the activation marker CD69 become highly up-regulated, while the expression of the lymph node homing molecule CD62L declines. This figure exemplarily shows the *ex vivo* IL-2 stimulated NK cell phenotype present in the fresh NK-DLI_IL-2stim_. Density plots show CD56 vs. CD16 (CD56-PE, gated on lymphocytes excluding CD3^+^ T cells and CD19^+^ B cells), CD56 vs. NKp44, CD56 vs. CD69 and CD56 vs. CD62L (CD56-PC7, gated on CD56^+^CD3^−^ NK cells). B) The NK cell phenotype of the NK-DLI_IL-2 stim_ (Fig. S3A) was not present at any time following NK-DLI_IL-2 stim_ application in patients' PB. This was indicated by the red circles. The blue squares highlight the CD62L expression of PB NK cells of the patients, illustrating the loss of the CD62L^+^ expressing PB NK cell phenotype as early as 10 min post NK-DLI_IL-2 stim_ infusions, and the recovering after 24 h. In addition, CD56 was not down-regulated after NK-DLI_IL-2 stim_ application. Density plots show CD56 vs. CD16 (CD56-PE, gated on lymphocytes excluding CD3^+^ T cells and CD19^+^ B cells), CD56 vs. NKp44, CD56 vs. CD69 and CD56 vs. CD62L (CD56-PC7, gated on CD56^+^CD3^−^ NK cells). C) The decline in CD62L expressing PB NK cells was further illustrated by a box and whiskers plot. In the PB nearly all of the minor CD56^bright^ and about 40% of the major CD56^dim^ NK cell subpopulation are CD62L^+^. As early as 10 min post NK-DLI_IL-2 stim_ applications (n = 4) a significant reduction in CD62L^+^ expressing PB CD56^+^CD3^−^ NK cells was seen, while returning after 24 h. p<0.05 is indicated as *.
(TIF)

Figure S4 **Impact of total NK-DLI_IL-2 stim_ applications on leukocyte subpopulations and cytokine/chemokine levels.** This figure gives an overview of all NK-DLI_IL-2 stim_ applications, fresh and cryopreserved, in total. All effects of freshly applied NK-DLI_IL-2 stim_ on leukocyte subpopulations shown in Fig. 1, 2, 3 were comparable to those presented in the overall of all NK-DLI_IL-2 stim_ applications (n = 13). Box and whiskers plots show minimum, lower quartile, median, upper quartile and maximum of all measured data. Cytokine analyses of PB plasma samples collected before (pre) and 10 min, 1 h, 4 h and 24 h after fresh and cryopreserved NK-DLI_IL-2 stim_ in total. Similar significant increases of i*n vivo* cytokine/chemokine concentration of IL-2, IL-6, IL-8, IFN-γ, MCP-1 and MIP-1β in patient's plasma compared to exclusively fresh NK-DLI_IL-2 stim_ applications (Fig. 3) were observed. Y-axis shows cytokine/chemokine concentration, range 1–1.000.000 pg/ml. p<0.05, p<0.01 and p<0.001 are indicated as *, ** and ***, respectively.
(TIF)
