# Supplementary figures and images for: IL-2 Stimulated but Not Unstimulated NK Cells Induce Selective Disappearance of Peripheral Blood Cells: Concomitant Results to a Phase I/II Study
Source: PLoS One. 2011 Nov 9;6(11):e27351. doi: 10.1371/journal.pone.0027351 (PMC3212563; doi:10.1371/journal.pone.0027351)

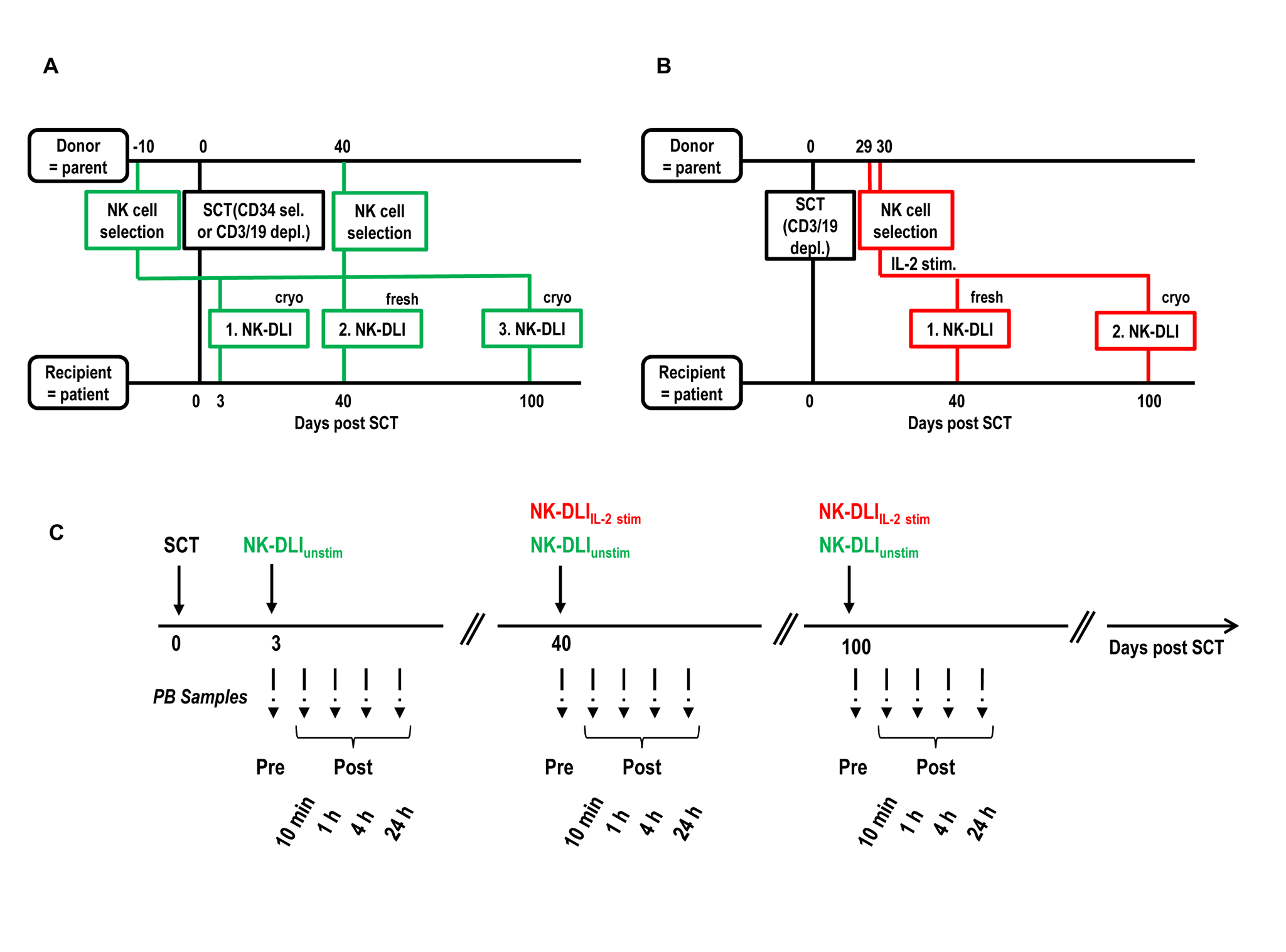

Supplement: Figure S1 — Study designs of the clinical phase I/II NK-DLI and our concomitant in vivo monitoring analyses. A) In a phase I/II clinical feasibility study starting in the year 2003, haploidentical donor NK cells were isolated from unstimulated leukapheresis and purified by a two-step CD3-depletion/CD56-selection procedure. For haplo-SCT (d 0), peripheral blood stem cells (PBSC) were purified immunomagnetically either by CD34-selection or CD3/CD19-depletion. For NK cell collection, leukapheresis was performed at day −10 prior and +40 post SCT. At day +40, NK-DLIunstim was applied freshly, directly at the end of the purification process, while the processed NK-DLIunstim from day −10 was split and cryopreserved for the NK cell application on day +3 and +100. B) In an amendment of the study starting in the year 2005 two leukapheresis products collected on day +29 and +30 post SCT were pooled for the CD3-depletion/CD56-selection NK cell purification process. Following purification, NK cells were further ex vivo expanded and activated using 1000 U/ml IL-2 for 10 (9 to 14) days obeying GMP. After ex vivo stimulation, the NK cell product was split up, while one half was infused freshly at day +40 and the other was cryopreserved and applied at day +100 post SCT. For haplo-SCT (d 0), peripheral blood stem cells (PBSC) were purified immunomagnetically by CD3/CD19-depletion. C) For our concomitant in vivo monitoring study during NK-DLI, PB samples were collected at the day of application before (pre), 10 min, 1 h, 4 h and 24 h after the end of NK-DLI application. (TIF) [file pone.0027351.s001.tif]

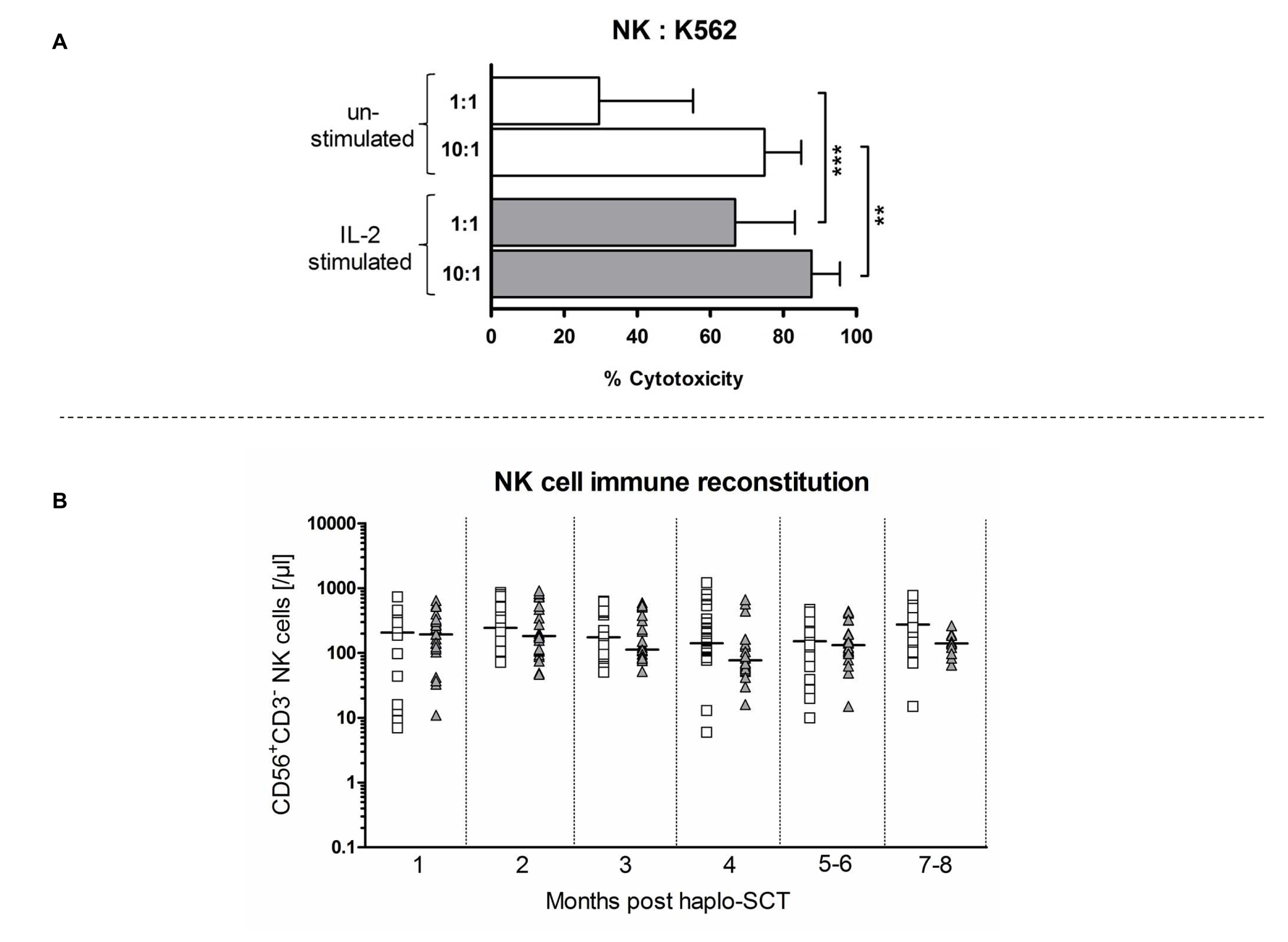

Supplement: Figure S2 — A) NK cell cytotoxicity of NK-DLIunstim vs. NK-DLIIL-2 stim. Cytotoxic activity of donor NK cells against K562 was significantly enhanced by IL-2 stimulation. The killing activity against the MHC class I negative leukemic cell line K562 of IL-2 stimulated NK-DLIs (grey, n = 9) was significantly greater compared to unstimulated NK cells (white, n = 9) at both effector∶target ratios 1∶1 and 10∶1. NK cell cytotoxicity of freshly isolated unstimulated or IL-2 stimulated products was tested previously to application to the patients and/or before cryopreservation. Cytotoxicity was analyzed based on a 5-color flow cytometric single platform assay [19] and defined as the loss of viable target cells in relation to the mono-cultured control. p<0.01 and <0.001 indicated as ** and ***. B) In vivo NK cell immune reconstitution post haplo-SCT. Similar NK cell immune reconstitution post haplo-SCT in both patients' groups receiving NK-DLIIL-2 stim and NK-DLIunstim respectively. Very similar NK cell immune reconstitution was seen in both patient subgroups NK-DLIunstim (□, n = 7) and NK-DLIIL-2 stim (▴, n = 6). Immune reconstitution of all patients was monitored regularly; within the first three months post SCT weekly, from month four to six twice a month, followed by a period of monthly analyses. Shown are all measurements and median performed in each interval which were similar in both groups. (TIF) [file pone.0027351.s002.tif]

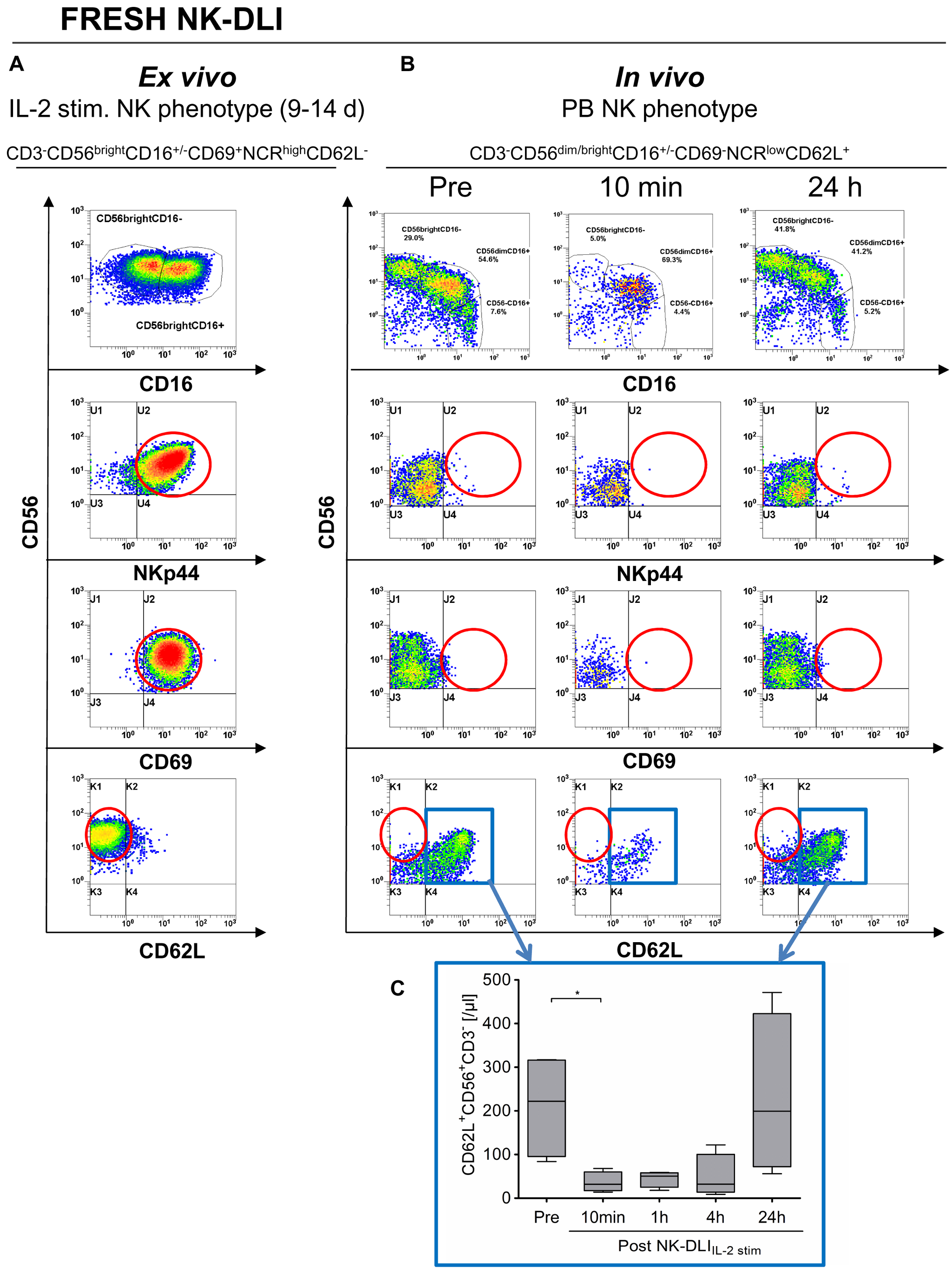

Supplement: Figure S3 — In vivo NK cell phenotype in patients PB differs from that of ex vivo expanded NK-DLIIL-2 stim. A) Upon 9–14 days of IL-2 stimulation, CD56, NKp44 and the activation marker CD69 become highly up-regulated, while the expression of the lymph node homing molecule CD62L declines. This figure exemplarily shows the ex vivo IL-2 stimulated NK cell phenotype present in the fresh NK-DLIIL-2stim. Density plots show CD56 vs. CD16 (CD56-PE, gated on lymphocytes excluding CD3+ T cells and CD19+ B cells), CD56 vs. NKp44, CD56 vs. CD69 and CD56 vs. CD62L (CD56-PC7, gated on CD56+CD3− NK cells). B) The NK cell phenotype of the NK-DLIIL-2 stim (Fig. S3A) was not present at any time following NK-DLIIL-2 stim application in patients' PB. This was indicated by the red circles. The blue squares highlight the CD62L expression of PB NK cells of the patients, illustrating the loss of the CD62L+ expressing PB NK cell phenotype as early as 10 min post NK-DLIIL-2 stim infusions, and the recovering after 24 h. In addition, CD56 was not down-regulated after NK-DLIIL-2 stim application. Density plots show CD56 vs. CD16 (CD56-PE, gated on lymphocytes excluding CD3+ T cells and CD19+ B cells), CD56 vs. NKp44, CD56 vs. CD69 and CD56 vs. CD62L (CD56-PC7, gated on CD56+CD3− NK cells). C) The decline in CD62L expressing PB NK cells was further illustrated by a box and whiskers plot. In the PB nearly all of the minor CD56bright and about 40% of the major CD56dim NK cell subpopulation are CD62L+. As early as 10 min post NK-DLIIL-2 stim applications (n = 4) a significant reduction in CD62L+ expressing PB CD56+CD3− NK cells was seen, while returning after 24 h. p<0.05 is indicated as *. (TIF) [file pone.0027351.s003.tif]

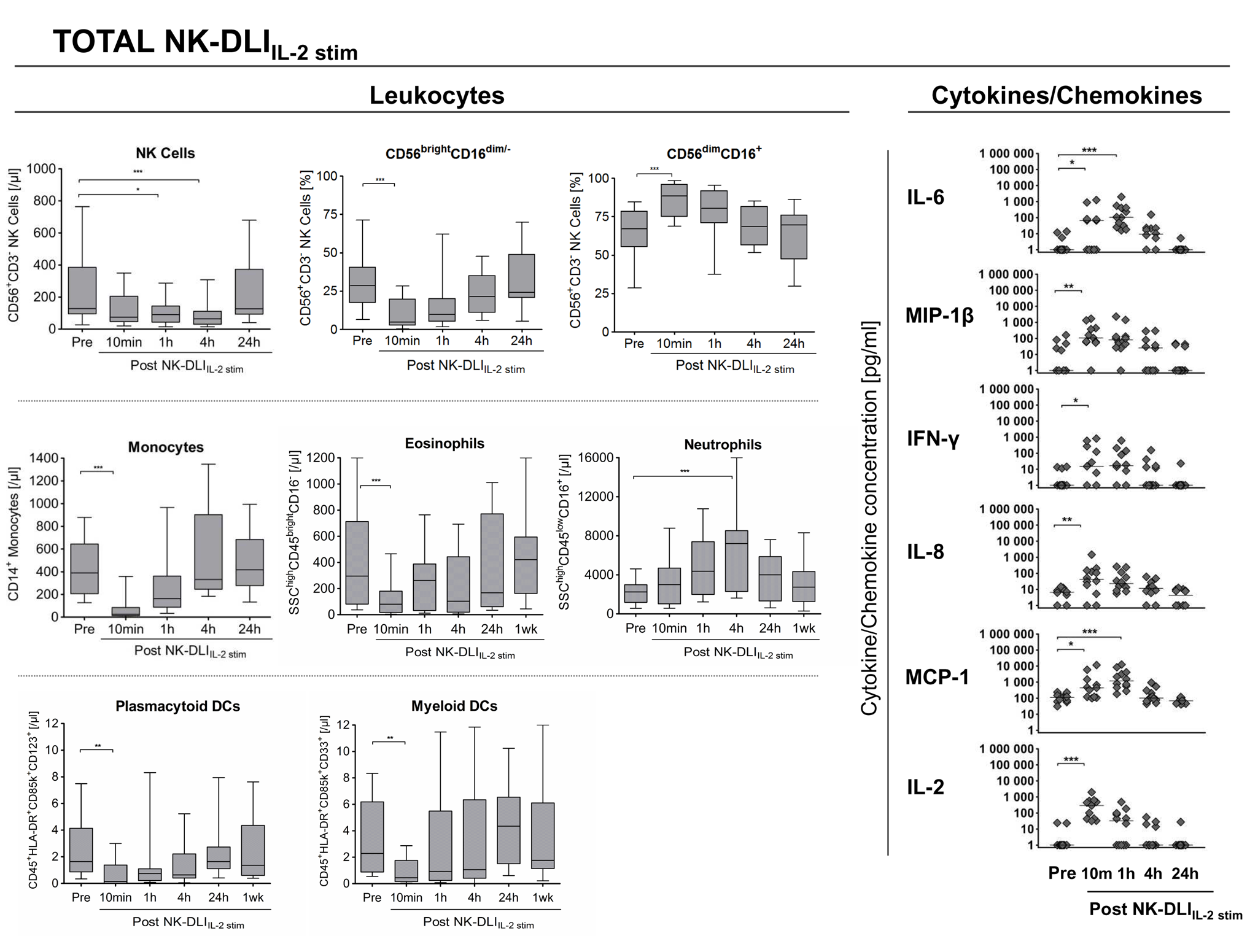

Supplement: Figure S4 — Impact of total NK-DLIIL-2 stim applications on leukocyte subpopulations and cytokine/chemokine levels. This figure gives an overview of all NK-DLIIL-2 stim applications, fresh and cryopreserved, in total. All effects of freshly applied NK-DLIIL-2 stim on leukocyte subpopulations shown in Fig. 1, 2, 3 were comparable to those presented in the overall of all NK-DLIIL-2 stim applications (n = 13). Box and whiskers plots show minimum, lower quartile, median, upper quartile and maximum of all measured data. Cytokine analyses of PB plasma samples collected before (pre) and 10 min, 1 h, 4 h and 24 h after fresh and cryopreserved NK-DLIIL-2 stim in total. Similar significant increases of in vivo cytokine/chemokine concentration of IL-2, IL-6, IL-8, IFN-γ, MCP-1 and MIP-1β in patient's plasma compared to exclusively fresh NK-DLIIL-2 stim applications (Fig. 3) were observed. Y-axis shows cytokine/chemokine concentration, range 1–1.000.000 pg/ml. p<0.05, p<0.01 and p<0.001 are indicated as *, ** and ***, respectively. (TIF) [file pone.0027351.s004.tif]
